# The radioanatomical assessment of the Körner’s septum

**DOI:** 10.1007/s00276-018-2149-3

**Published:** 2018-12-11

**Authors:** Tomasz Wojciechowski, Tymon Skadorwa, Adrian Drożdż, Bogdan Ciszek, Kazimierz Szopiński

**Affiliations:** 1 Department of Descriptive and Clinical Anatomy, The Medical University of Warsaw, 5 Chalubinskiego St, 02004 Warsaw, Poland; 2 Department of Otolaryngology, The Medical University of Warsaw, 1a Banacha St, 02097 Warsaw, Poland; 3 Department of Pediatric Neurosurgery, Bogdanowicz Memorial Hospital for Children, 4/24 Nieklanska St, 03924 Warsaw, Poland; 4 Department of Dental and Maxillofacial Radiology, The Medical University of Warsaw, 59 Nowogrodzka St, 02006 Warsaw, Poland

**Keywords:** Körner’s septum, Petrosquamous lamina, Temporal bone, Cone-beam CT

## Abstract

**Purpose of the study:**

Körner’s septum (KS) is a developmental remnant formed at the junction of mastoid and temporal squama, representing the persistence of the petrosquamosal suture. During mastoid surgery, it could be taken as a false medial wall of the antrum so that the deeper cells might not be explored. The aim of the study was to assess a Körner’s septum prevalence and to analyze its topography.

**Methods:**

The study was performed on 80 sets of cone-beam computed tomography (CBCT) images of temporal bone (41 male, 39 female, 160 temporal bones). Körner’s septum was identified and its thickness was measured on axial sections at three points: at the level of superior semicircular canal (SCC), at the level of head of malleus (HM) and at the level of tympanic sinus (TS).

**Results:**

KS was encountered at least in one point of measurements in 50 out of 80 sets of CBCT images (62.5%). The average thickness at the level of SCC was 0.87 ± 0.34 mm, at the level of HM was 0.99 ± 0.37 mm and at the level of TS was 0.52 ± 0.17 mm.

**Conclusions:**

Körner’s septum is a common structure in the temporal bone–air cell complex. It is more often encountered in men. In half of the patients, it occurs bilaterally. However, in most of the cases it is incomplete with anterior and superior portions being the most constant.

## Introduction

Körner’s septum (KS) also known as petrosquamous lamina is a bony plate formed at the junction of the mastoid and temporal squama and it represents the persistence of the petrosquamous suture [17, 21]. It does not only divide the antrum into petrous (deep) and squamous (superficial) portions [17], but it also descends toward the mastoid process posteriorly, and ascends above the air spaces of the middle ear to finally reach the posterior aspect of the mandibular fossa [5, 17].

Despite the fact that some authors point to A. H. Cheatle as the first to describe KS [5, 17] using his memorable Hunterian Lectures as a proof [3], it was actually A. Hartmann who brought it to otologists’ attention nearly 20 years earlier. In his work, he wrote about a large cavity in the temporal bone—antrum petrosum—that is partly separated from antrum mastoideum by the osseus plate projecting from the tegmen [7].

Since Körner highlighted the clinical relevance of the septum [9], the importance of its morphology has been almost unnoticeable in the literature [19]. After William House first used microscope to perform temporal bone dissection in 1958, Shulman and Rock published a paper in which they stated that a well-developed KS may be confused for the bony covering of the sigmoid sinus [15]. Consequently, the false antrum may be entered, or the facial nerve can even be injured [10]. Since then, diagnostic imaging of the temporal bone significantly evolved, especially after introduction of computed tomography (CT) in early 70s. It became a lot easier to study KS and such a fact was immediately availed by a few researchers, e.g., Virapongse et al. [19, 21]. Currently, high-resolution computed tomography (HRCT) is used in preoperative surgical planning of the temporal bone [17]. Lately, a few reports have described using cone-beam CT in otosurgical diagnostics as an excellent alternative for HRCT with lower radiation doses [8]. It may be used in the evaluation of anatomic variations, including the presence of Körner’s septum, and evaluation of pneumatization of the mastoid [1, 2, 4, 6, 14, 20].

Although some authors mentioned the KS in their papers, no papers describing its structure have been published to our knowledge. Therefore, we aim to assess in our study the Körner’s septum prevalence and analyze its topography.

## Materials and methods

The study was performed on anonymized sets of cone-beam computed tomography (CBCT) images of temporal bone gathered from the Department of Dental and Maxillofacial Radiology, Medical University of Warsaw, between February 2013 and June 2013. All the scans were obtained due to clinical (dental or maxillofacial) indications with the scanner Planmeca Promax 3D Mid (Planmeca USA, INC, Roselle, Illinois, USA) with parameters: voxel dimensions 400 × 400 × 400 µm; the exposition was performed with source voltage of 90 kV and current of 12 mA. From the initial group (141 patients), the images with any temporal bone pathology were excluded (61 patients). Thus, a final group of 80 sets of images (41 male and 39 female, 160 temporal bones) was enrolled to the study. The age of the patients involved in the analysis ranged from 12 to 77 (mean age 37 ± 16 years).

In the next phase, the scans were analyzed in RadiAnt DICOM Viewer 4.0.3 (64-bit), obtained data were analyzed statistically with the use of StatSoft Statistica 13.1 software.

**Fig. 1:**
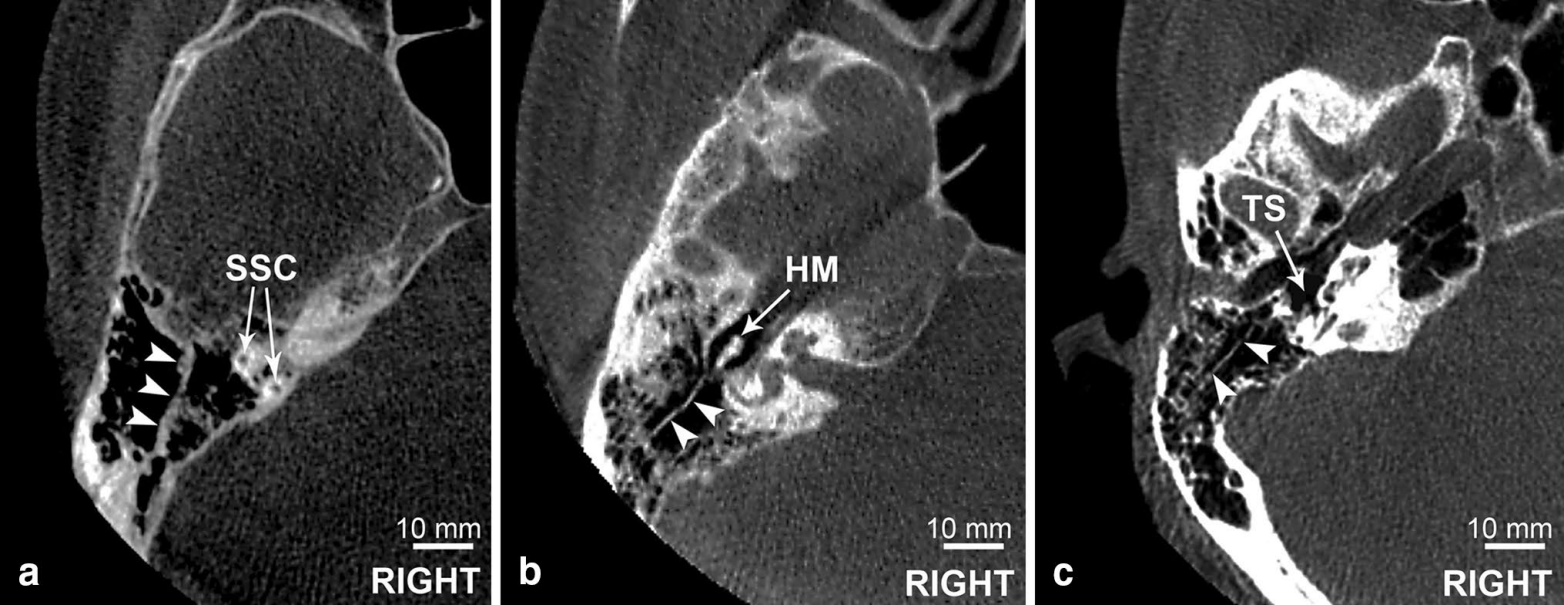
Axial CBCT scans presenting all the three landmarks used in the study. KS marked with arrowheads. **a***SSC* superior semicircular canal; **b***HM* head of malleus; **c***TS* tympanic sinus

**Fig. 2:**
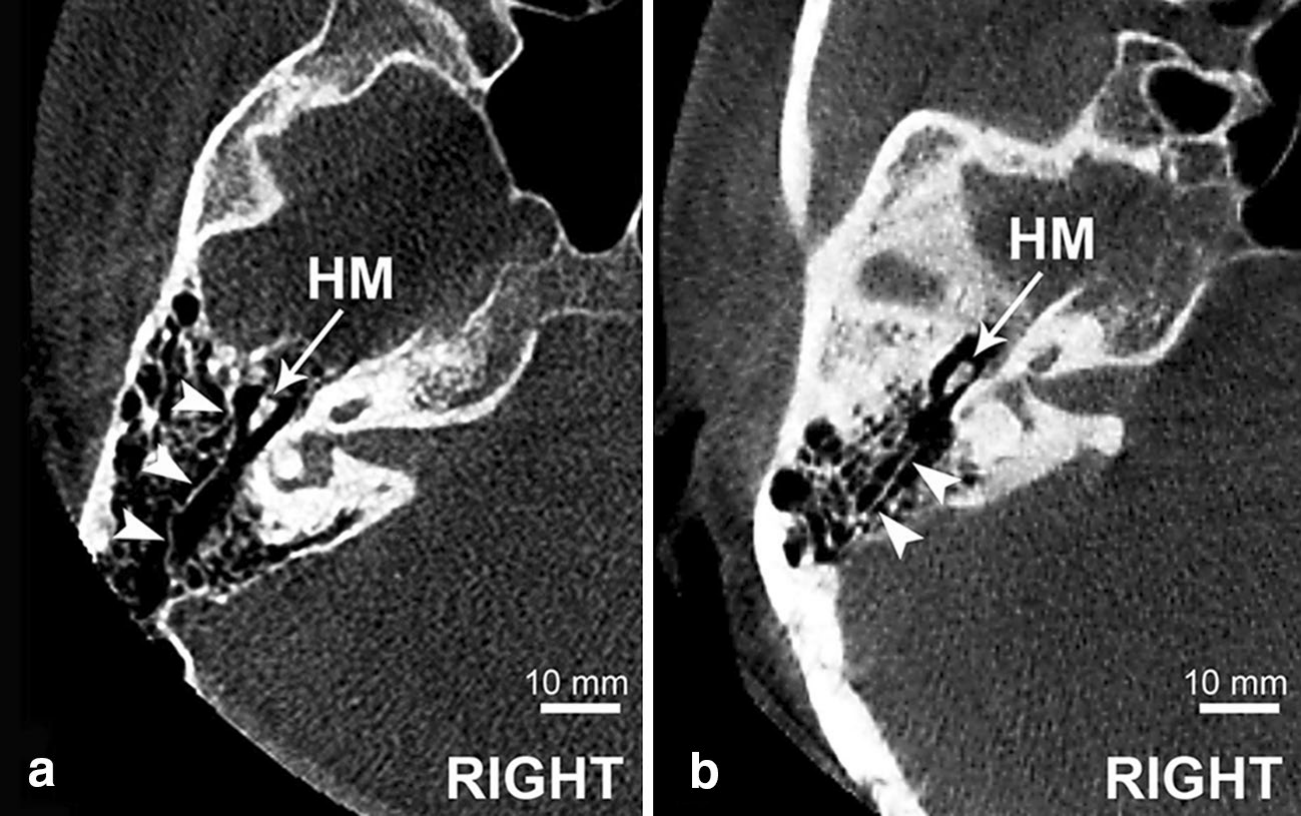
Axial CBCT scans at the level of HM presenting complete (**a**) and incomplete KS (**b**). KS marked with arrowheads

The subsequent step was to identify the Körner’s septum and to measure its thickness on horizontal sections at three points (landmarks): at the level of superior semicircular canal (SSC), at the level of head of malleus (HM) and at the level of tympanic sinus (TS) (Fig. 1). The KS was classified as present when there was a bony lamina encountered in at least one of the points mentioned above. The presence of KS was also categorized as complete, when septum consisted of all three portions, and incomplete, when one or two portions were missing (Fig. 2).

## Results

We were able to find all anatomical landmarks (HM, SCC and TS) for every part of Körner’s septum on all evaluated CBCT scans. KS was recognized at least in one point of measurements in 50 out of 80 sets of CBCT images (62.5%), which constituted 75/160 temporal bones. The KS was more frequent in males than females (71% vs 53%; *p* > 0.05) and it was present bilaterally in half of the cases (25 people out of 50). A complete KS, present at all three points, was encountered in 19 out of 75 temporal bones (25%) but it was found bilaterally only in 3 subjects—2 women and 1 man, which made 5% and 2% of our population, respectively. An incomplete KS was encountered in 56/75 temporal bones (75%). The name for each portion of KS was adopted from anatomical directions, taking into consideration the position of KS in the space of the antrum. Therefore, the level of SCC corresponded to superior portion of KS, the level of HM to anterior portion and the level of TS to its posterior portion.

KS was present most commonly at the level of head of malleus—it was identified at this point 54/160 cases (34%)—32 in males (39%) and 22 in females (28%). KS was recognized at the level of superior semicircular canal in 53/160 cases (33%)—29 males (35%) and 24 females (31%). At the last point (sinus tympani), KS was found in 31 bones (19%)—16 males and 15 females (19% each).

The average thickness of KS was significantly different in every portion (K–W test; *p* < 0.05). It was the greatest at the level of HM and the lowest at the level of TS. The average thickness with standard deviation, median, minimal and maximal values of thickness are presented in Table 1.

**Table 1:** Thickness of KS at measured levels (*n* number of cases; *avg* average value; *SD* standard deviation; *SSC* superior semicircular canal; *HM* head of malleus; *TS* tympanic sinus)

	SSC (mm)	HM (mm)	TS (mm)
*n*	53 (33%)	54 (34%)	31 (19%)
Avg ± SD	0.87 ± 0.35	1.00 ± 0.38	0.53 ± 0.17
Median	0.73	0.93	0.51
Min–max	0.28–1.74	0.26–2.60	0.19–0.78

## Discussion

### Results’ discussion

In spite of the fact that Körner’s septum has been known to anatomists and otologists for more than 100 years [3, 7], it is striking that there are no reports about its detailed morphometry and variant classification.

Proctor et al. stated that complete or partial persistence of KS is a result of a failure during development of the mastoid air cell system [12]. On the other hand, a few authors point that KS does not occur only at the level of the antrum but it may consist of at least one of three portions [5, 10, 12, 13, 21].

There are many discrepancies and inaccuracies in case of naming each part of KS. According to Proctor et al., there are three parts of the squama and as a consequence, three points of junction with the pars petrosa: (1) starting from the anterior edge of temporomandibular joint and forming its roof, (2) through the superior wall of external acoustic meatus to the (3) mastoid or posterior part overlaying a great part of the petrous bone. They also described the internal petrosquamous suture positioned anterosuperiorly to the tympanic sulcus (anterior internal petrosquamous suture) and the posterior external petrosquamous suture which represented the contact of the deep aspect of the retromeatal portion of the squama with the pars petrosa. Interestingly, they claimed that structure can be called a Körner’s septum or petrosquamous lamina when the junction of the pars petrosa and squama persists only inside the mastoid process [12]. The same structures have been described by Virapongse et al., but different terminology was used. Ventral (temporomandibular) petrosquamosal suture was found ventrolateral to the protympanum and Eustachian tube isthmus. The mid- or tympanic portion of petrosquamous suture was classified as very difficult to find in axial images in the region of epitympanum. Possibly it may look like a bony spicule above the ossicular chain, but it has not been definitely proven [21]. In our study, we did not encounter any structure in axial and coronal images that could correspond to structures defined like mentioned above. On the other hand, we have found that the superior portion of the attic outer wall is continuous with the Körner’s septum inside the mastoid process and that structure was described in our study as the anterior portion of KS. And then, there is the last portion of KS described by Virapongse et al.—dorsal or mastoid part in which they distinguish the superior and inferior part. Attention is brought to the fact that KS does not have to be complete—it can be interrupted in more than one place [19, 21]. The following question has been raised—is this due to some kind of developmental arrest or bony destruction in a result of a disease? No detailed classification has been proposed yet. What is more, there is no agreement about the structure known as ‘the cog’—some authors state that this is the same part as tympanic or mid-portion of KS [10]. On the other hand, Tóth describes a small bony plate extending from tignum transversum anteriorly to the Glaserian fissure and then breaking medially between anterior malleal space and anterior epitympanic recess [18] which he has also called ‘the cog’.

In our study, we have chosen three areas in which we expected to find the Körner’s septum. At the level of tympanic sinus, in close relationship with a mastoid part of facial canal, we have searched for posterior portion of KS. Superior semicircular canal was a landmark for the superior portion of the KS. This one can also occur as the only persistent part of KS which can lead an otosurgeon straightforward to the posterior crus of the incus [13]. In certain cases, the superior part extends anteriorly and fuses with the outer attic wall—we call it the anterior part of the KS—it can be identified on CBCT scans in both axial and coronal planes.

According to the literature, the presence of Körner’s septum may vary in different groups from approximately 6.5% [5] to even 45% [1] in temporal bones without a history of aural pathology. The second value is comparable to our result (47% of temporal bones). Körner’s septum was found in 28% of 356 ears that underwent tympanoplasty by Cigdem et al. [4]. Toros et al. under similar conditions found that KS was present in nearly 24% of the operated temporal bones [17]. Körner’s septum was encountered in about 21% of temporal bones studied by Goksu et al. [5] but it was more frequent (30.4%) in ears with chronic otitis media than in normal ears (6.58%). Additionally, Goksu et al. found that KS was incomplete in 33% which means that at least one part of it was missing. This is different from our results—we found a complete septum in only 25% of examined temporal bones. Our other results cannot be confronted with the literature, as no studies analyzing the possible association between the presence of KS and genders or sides have been published to our knowledge.

None of the published papers comments on the portions of KS and their dimensions. In the present study, we found that KS is remarkably thinner at the level of TS than in other portions (*p* < 0.05). The thickness of KS at the level of SSC is the same in males and females. The thickness at the level of HM is greatest than in other portions.

### Methodology discussion

Cone-beam CT was introduced in mid 90 s and has been used, especially in dental and maxillofacial radiology. Although high-resolution CT is the gold standard in the imaging of the temporal bone, there is an increasing number of papers that report attempts to use CBCT in otorhinolaryngology [8, 22] and imaging of the fine structures inside the temporal bone [11], particularly when there is need for preoperative assessment of cochlear implantation, or postoperative follow-up. Cone-beam CT technique is still developing and it seems to be a promising method for the diagnostics of chronic otitis media, or traumatic lesions. What is more important, it can be offered to children due to low dose of radiation and rapid acquisition with high spatial resolution [8]. However, when interpreting CBCT images one must remember that radiodensity cannot be measured reliably in Hounsfield units [16].

For this reason, the use of CBCT may be one of the limitations of this study. The use of micro-CT might possibly improve the spatial resolution of images, but the studies based on this technique usually include smaller samples, and this method cannot be used for studying the temporal bone anatomy in living subjects.

Another possible limitation was the non-availability of clinical data. All examinations were performed for dental or maxillofacial indications. As the data had been anonymized prior to enrollment to the study, we were not able to collect the history of any ear diseases in examined patients. Only the images with visible pathologies could have been excluded from the studied population. This fact, however, limits the possibility of discussion with other papers in terms of clinical observations.

## Conclusions

1. Körner’s septum is a common structure in the temporal bone–air cell complex. It is more often encountered in males.
2. Körner’s septum is composed of three portions: anterior (the most constant, identified at the level of head of malleus), superior (identified at the level of superior semicircular canal) and posterior (the least constant, at the level of tympanic sinus).
3. The thickness of the Körner’s septum is the greatest in anterior and the lowest in its posterior portion.
